# Role of Polyphosphate as an Inorganic Chaperone to Prevent Protein Aggregation Under Copper Stress in *Saccharolobus solfataricus*

**DOI:** 10.3390/microorganisms12122627

**Published:** 2024-12-18

**Authors:** José Acevedo-López, Gabriela González-Madrid, Claudio A. Navarro, Carlos A. Jerez

**Affiliations:** Laboratory of Molecular Microbiology and Biotechnology, Department of Biology, Faculty of Sciences, University of Chile, Santiago 7800003, Chile; jacevedol.1994@gmail.com (J.A.-L.); gabriela.gonzalez.m@ug.uchile.cl (G.G.-M.);

**Keywords:** *Saccharolobus solfataricus*, polyphosphate, inorganic molecular chaperone, copper stress, protein precipitation

## Abstract

Polyphosphates are biopolymers composed of phosphate monomers linked by high-energy phosphoanhydride bonds. They are present across all life domains, serving as a source of energy, metal chelators, and playing a crucial role in stress defense. In *Escherichia coli*, polyphosphates also function as inorganic molecular chaperones. The present study aims to investigate whether polyphosphate serves a similar chaperone function in archaea, using *Saccharolobus solfataricus* as a model organism. To this end, polyphosphate was extracted and quantified, the ADP/ATP ratio was determined, insoluble protein extracts were analyzed at different time points after copper exposure, and qPCR was performed to measure the expression of stress-related genes. PolyP was extracted after exposing the archaeon *S. solfataricus* to different copper concentrations. We determined that polyP degradation is directly correlated with metal concentration. At the minimum inhibitory concentration (MIC) of 2 mM Cu^2+^, polyP degradation stabilized 2 h after exposure and showed no recovery even after 24 h. The ADP/ATP ratio was measured and showed differences in the presence or absence of polyP. The analysis of proteins precipitated under copper stress showed a higher proportion of insoluble proteins at an elevated metal concentration. On the other hand, increased protein precipitation was detected in the absence of polyP. Gene expression analysis via qPCR was conducted to assess the expression of genes involved in chaperone and chaperonin production, copper resistance, oxidative stress response, and phosphate metabolism under prolonged copper exposure, both in the presence and absence of polyP. The results indicated an upregulation of all the chaperonins measured in the presence of polyP. Interestingly, just some of these genes were upregulated in polyP’s absence. Despite copper stress, there was no upregulation of superoxide dismutase in our conditions. These results highlight the role of polyP in the copper stress response in *S. solfataricus*, particularly to prevent protein precipitation, likely due to its function as an inorganic chaperone. Additionally, the observed protein precipitation could be attributable to interactions between copper and some amino acids on the protein structures rather than oxidative stress induced by copper exposure, as previously described in *E. coli*. Our present findings provide new insights into the protective role of polyP as an inorganic chaperone in *S. solfataricus* and emphasize its importance in maintaining cellular homeostasis under metal stress conditions.

## 1. Introduction

Polyphosphates (polyP) are biopolymers found across all living organisms, spanning the three domains of life. These polymers consist of orthophosphate units (Pi) linked by high-energy phosphoanhydride bonds [1,2,3,4,5]. PolyP chains are synthesized by the enzyme polyphosphate kinase (PPK) through a reversible reaction [1,2,3,4,5,6]. Conversely, Polyphosphate Exopolyphosphatase (PPX) mediates the irreversible hydrolytic degradation of the polymer [1,2,3,4,5,6,7]. In archaea, the first enzyme identified as involved in polyP metabolism was Polyphosphate Exopolyphosphatase (PPX) from *Saccharolobus solfataricus* [7]. Conversely, the enzyme responsible for polyP synthesis in Crenarchaeota was recently characterized in *Sulfolobus acidocaldarius* by Hofmann et al., 2024 [8].

PolyP has been implicated in various cellular functions. In bioleaching microorganisms, polyP serves crucial roles such as cation chelation and stress protection [1,9,10,11,12,13]. In bacteria, polyP has also been implicated in the stress response, such as nutrient starvation, participating in copper release via the PitA transporter, and as an inorganic molecular chaperone [1,2,4,14,15,16,17,18,19,20,21,22,23,24,25,26].

The precise mechanism by which copper enters *S. solfataricus* cells remains unclear; however, nonspecific secondary transporters have been proposed as potential entry pathways [11,27,28]. Once inside the cell, copper induces the formation of reactive oxygen species (ROS) via a Fenton reaction, resulting in macromolecular damage, the inactivation of Fe-S clusters, and the disruption of metabolic pathways due to protein damage. Consequently, efficient copper expulsion mechanisms are critical to mitigate these effects [4,29,30,31,32]. *S. solfataricus* employs two primary systems to cope with copper stress. The Cop system, which consists of ATPases (CopA and CopB), actively expels Cu^+^ through ATP hydrolysis. CopA is expressed at various Cu^2+^ concentrations, while CopB appears to be constitutive and less specific [28,33]. Additionally, the secondary transporters PitA and Pho84 have also been implicated in copper expulsion in Sulfolobales [9,11,13,27,28].

Copper-induced reactive oxygen species (ROS) can denature proteins by disrupting their tertiary structures [34,35]. To prevent the aggregation of denatured proteins, organisms utilize chaperones and chaperonins [36,37,38,39,40,41,42,43,44,45,46]. These molecular systems bind to exposed hydrophobic regions of unfolded proteins, thereby preventing aggregation [39,41,43,45,46]. The key distinction between these systems is that chaperonins, unlike chaperones, can actively restore the native conformation of proteins through ATP-dependent mechanisms [39,42,43,44,45,46]. Amongst the chaperones in *S. solfataricus*, members of the small heat shock proteins (sHSPs) superfamily and prefoldins can be found [37,40,43,47]. sHSPs are a superfamily of stress-inducible chaperones ranging from 12 to 43 kDa that form oligomers [40,47]. On the other hand, prefoldins consist of two subunits that assemble into a hexamer, which binds to denatured proteins and transfers them to the thermosome. The thermosome, a Group II chaperonin in *S. solfataricus*, is composed of two ring structures that form an oligomeric complex, consisting of up to three different subunits, responsible for ATP-dependent protein refolding [43,45,46,48,49,50].

Interestingly, it has been suggested that copper-induced protein damage can occur not only in aerobic environments but also in anaerobic conditions. This damage is likely facilitated by copper interacting with histidine and cysteine residues on the protein’s three-dimensional surface [51].

As previously mentioned, one of the key roles of polyP is its function as an inorganic molecular chaperone [4,16,17,18,19,20,21,22,23,24,25,26]. In 2014, Gray et al. found that polyP acts as a chaperone in *E. coli* under oxidative stress, maintaining proteins in a “refolding-competent” state by stabilizing them in a β-sheet-rich conformation [4,16,17,18,19,20,21,22,23,24,25,26]. However, this chaperone role of polyP has not been previously described in archaea. Investigating whether polyP of *S. solfataricus* functions as an inorganic molecular chaperone could offer significant insights into its role in maintaining protein homeostasis in archaea.

In our laboratory, we use two strains of *S. solfataricus*, M16 and M16-PPX. The latter strain contains an additional copy of the *ppx* gene, which is inducible by D-arabinose. After 3 h of induction, the M16-PPX strain lacks polyP [12]. Using these strains, we explored the potential role of polyP as an inorganic molecular chaperone under copper stress conditions. PolyP degradation at different copper concentrations and the ADP/ATP ratio due to its association with polyP metabolism were investigated. Additionally, protein precipitation and the expression of stress-related genes in *S. solfataricus*, such as chaperonins, chaperones, copper resistance proteins, polyP metabolism enzymes, and oxidative stress detoxifiers, were also explored. These analyses were conducted under copper stress in both the presence and absence of polyP.

Our study reveals that polyP plays a crucial role in the response of *S. solfataricus* to copper-induced stress, being important for preventing protein aggregation, possibly due to its function as an inorganic chaperone. Nevertheless, its role as an energy source could also contribute to its protective effects, as polyP degradation is closely tied to energy metabolism, influencing the ADP/ATP ratio. This dual role of polyP—as both a molecular chaperone and an energy reserve—highlights its multifaceted importance under metal-induced stress conditions. To our knowledge, our group is the first to investigate the function of polyP as an inorganic chaperone in archaea, providing novel insights into its role in *S. solfataricus*.

## 2. Materials and Methods

### 2.1. Strains and Culture Media

*S. solfataricus* strains M16 (pyrEF mutant, auxotrophic for uracil) and M16-PPX (complemented for pyrEF and containing a copy of *S. solfataricus ppx* gene inducible by D-arabinose) were used in this study [12].

Both strains were grown at 75 °C with shaking at 120 rpm in Brock medium (1.3 g/L (NH_4_)_2_SO_4_, 0.28 g/L KH_2_PO_4_, 0.25 g/L MgCl_2_ × 7 H_2_O, 0.07 g/L CaCl_2_ × 2 H_2_O, 0.02 g/L FeCl_2_ × 4 H_2_O, 1.8 mg/L MnCl_2_ × 4 H_2_O, 4.5 mg/L Na_2_B_4_O_7_ × 10 H_2_O, 0.22 mg/L ZnSO_4_ × 7 H_2_O, 0.06 mg/L CuCl_2_ × 2 H_2_O, 0.03 mg/L Na_2_MoO_4_ × 2 H_2_O, 0.03 mg/L VOSO_4_ × 2 H_2_O and 0.01 mg/L CoCl_2_ × 6 H_2_O), pH 3 supplemented with 0.1% (*w*/*v*) N-Z amine (Sigma-Aldrich^®^, St. Louis, MO, USA), 0.2% (*w*/*v*) sucrose, and with 0.01 mg/mL uracil only in the case of the M16 strain. The recombinant PPX production was induced by adding 0.2% (*w*/*v*) D-arabinose (Sigma-Aldrich^®^, St. Louis, MO, USA).

### 2.2. polyP Extraction and Measurement

PolyP extraction was performed as previously described [12]. Cells were washed twice with basal Brock medium pH 5, centrifuged at 4000× *g*, resuspended in resuspension buffer (4 M GITC, 50 mM Tris-HCl pH 7.0), and lysed heating at 95 °C for 5 min. A total of 30 μL of 10% SDS was added, and samples were heated for 2 min. At this point, 20 μL was taken to measure the protein concentration using Bradford Reagent (Sigma-Aldrich^®^) and Infinite^®^ M Nano (Tecan Trading AG ©, Männedorf, Switzerland) according to the manufacturer’s instructions. A total of 300 μL of 50% ethanol and 5 μL of 15% Glass Milk were added, reheated at 95 °C for 30 s, and centrifuged at 13,000× *g* for 1 min. Pellets were resuspended in 200 μL of New Wash Buffer (5 mM Tris-HCl pH 7.5, 50 mM NaCl, 5 mM EDTA, 50% ethanol) and centrifuged at 13,000× *g* for 30 s. Pellets were resuspended in 86 μL of molecular-grade water, 1 μL of DNase (RNase-free) (New England Biolabs^TM^, Ipswich, MA, USA), and 3 μL of RNase A (New England Biolabs^TM^) and incubated at 37 °C for 30 min. After that, the samples were rewashed with New Wash Buffer, and finally, polyP was released in two rounds of washing with 100 μL of molecular-grade water and heating for 2 min at 95 °C.

To measure polyP levels, 30 μL of the samples was treated with 30 μL of 1 M HCl for 30 min at 95 °C. Samples were cooled at room temperature, and 60 μL of Tris-HCl 1.5 M pH 10 was added. The quantification was performed with the EnzChek^TM^ Phosphatase Assay kit (Invitrogen^TM^, Waltham, MA, USA) according to the manufacturer’s instructions. Finally, the PolyP levels were normalized by the amount of proteins.

### 2.3. ADP/ATP Assays

Cells were grown until early stationary phase (0.8–0.9 OD_600 nm_) and were supplemented with 0.2% D-arabinose. After 3 h of induction, 2 mM of CuSO_4_ was added and aliquots were taken at different points after stress. Cells were collected by 10 min centrifugation at 4000× *g* and cells were washed 3 times with Brock medium pH 5. To measure the ADP/ATP ratio, the ADP/ATP Ratio Assay kit (Sigma-Aldrich^®^), St. Louis, MO, USA) was used according to the manufacturer’s instructions.

### 2.4. Protein Extraction, Measurement, and Visualization

To study protein aggregation due to copper stress, cells were grown until they reached early stationary phase (0.8–0.9 OD_600 nm_) and were supplemented with 0.2% D-arabinose. After 3 h of induction, 2 mM of CuSO_4_ was added, and 15 mL aliquots were taken at different times. Cells were collected by 10 min centrifugation at 4000× *g*. After that, cells were washed 3 times with Brock medium pH 5 and resuspended in a sonication buffer (50 mM Tris–HCl pH 8.5, 10 mM EDTA, PMSF 100 μg/mL). Cells on ice were then disrupted by sonication (Sonicator Ultrasonic Processor, QSONICA^®^, Newtown, CT, USA) using 5 cycles of 20 s pulses with 30 s pauses in between. The lysates were then centrifuged at 7000× *g* to eliminate cellular debris. Supernatants were centrifuged for 30 min at 21,000× *g* to separate insoluble (pellets) and soluble (supernatants) proteins. Pellets were then resuspended in Tris-HCl buffer (Tris–HCl pH 8.5, 10 mM EDTA, 1% Nonidet P40), and 30 μL was taken to measure the protein concentration. The rest of the volumes were boiled for 2 min at 95 °C with 4X Laemmli Sample Buffer (Bio-Rad ©, Hercules, CA, USA) and stored at −20 °C until they were visualized by SDS-PAGE.

The protein concentrations were measured using Bradford Reagent (Sigma-Aldrich^®^) and Infinite^®^ M Nano (Tecan Trading AG ©, Männedorf, Switzerland) according to the manufacturer’s instructions. The ratio between insoluble and total protein was determined as the amount of insoluble proteins divided by the sum of the amount of soluble and insoluble proteins.

### 2.5. Total RNA Extraction from S. solfataricus and cDNA Synthesis

To study the expression of the genes of interest, cells were grown until they reached the early stationary phase (0.8–0.9 OD_600 nm_) and were supplemented with 0.2% D-arabinose. After 3 h of induction, 2 mM of CuSO_4_ was added and the cells were collected (20 mg wet weight) at different times and lysed as previously described [12]. RNA was extracted by using TRIzol (Invitrogen^®^) as described by the manufacturer. The remaining DNA was eliminated by adding 1 μL of DNase I (RNase-free) (New England Biolabs^TM^). A total of 50 ng of the total RNA was reverse-transcribed for cDNA synthesis. The reaction was performed by using the AffinityScript QPCR cDNA Synthesis Kit (Agilent ©, Santa Clara, CA, USA). Three biological replicates were used, with two technical replicates per reaction.

### 2.6. Primer Design and qPCR

Primers were designed using the annotated genome of *S. solfataricus* P2, Primer3 Software (Version 4.1.0), and SnapGene Viewer (Version 7.2). The primers used are listed in Table S1. The primers were synthesized by Invitrogen. To standardize the optimal melting temperature and primer concentrations, PCR reactions were carried out using Taq DNA Polymerase (New England Biolabs^TM^) following the manufacturer’s instructions. The products obtained were visualized by using gel electrophoresis (1% *w*/*v* agarose). Gene expression was analyzed with the Corbett Rotor Gene 6000 system (Corbett Life Sciences/Quiagen company, Hilden, Germany) using 10 μL of Forget-Me-Not^TM^ EvaGreen^Ⓡ^ qPCR Master Mix (Biotium, Inc., Fremont, CA, USA), 1 μL of diluted cDNA, and between 0.1 and 0.4 nM of primers Fw and Rv, according to the previous primer standardization. 23S was selected as a housekeeping gene and Cq values of each transcript of interest were standardized to the Cq value of 23S. The efficiency of each primer was calculated as in Soto et al., 2018 [12]. Efficiencies between 90 and 110% were used. Cq values (quantification cycle) were automatically determined by Real-Time Rotor-gene 6000 PCR software (Corbett Life Sciences/Quiagen company, Hilden, Germany) after 40 cycles. Cq values of each transcript of interest were standardized to the Cq value of the 23S gene. At least 3 biological replicates of each assessed condition and 2 technical replicates per qPCR reaction were performed.

### 2.7. Statistical Analyzes

Graphs and statistical analyses were performed using GraphPad Prism 10 (GraphPad Software Inc., San Diego, CA, USA). A two-way ANOVA with multiple comparisons was applied to identify differences between groups. When significant differences were detected, post hoc tests were used: Tukey’s test for independent group comparisons and Bonferroni’s test for paired comparisons.

The significance thresholds were set as follows: * = *p* ≤ 0.05, ** = *p* ≤ 0.01, *** = *p* ≤ 0.001, and **** = *p* ≤ 0.0001 (Bonferroni’s test); # = *p* ≤ 0.05, ## = *p* ≤ 0.01, ### = *p* ≤ 0.001, and #### = *p* ≤ 0.0001 (Tukey’s test). Non-significant differences were indicated as ns.

All analyses were performed with at least three biological replicates. Data are presented as the mean ± standard deviation. The selection of post hoc tests was based on the nature of the comparisons to ensure the appropriate statistical interpretation of the results.

## 3. Results

### 3.1. Polyphosphate Levels in the Presence of Copper Stress in S. solfataricus

**Figure 1:**
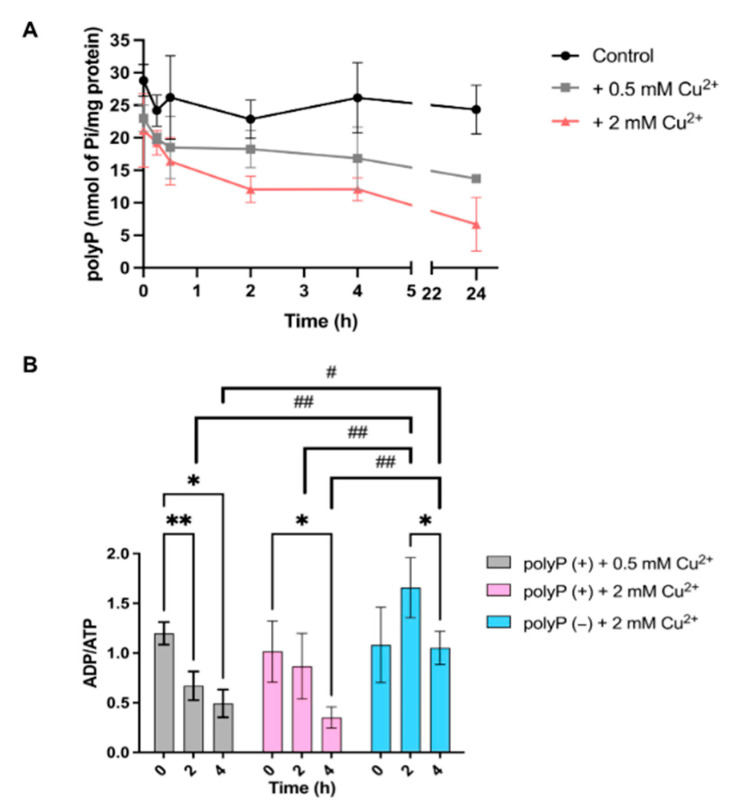
Polyphosphate degradation in *S. solfataricus* M16 (polyP+) and ADP/ATP ratio during copper stress in the presence (M16) or absence of polyP (M16-PPX). (**A**) Colored lines represent polyP levels measured at different time points following exposure to varying Cu^2+^ concentrations. Measurements represent the average of three biological replicates, with error bars indicating standard deviations. (**B**) In gray and pink is the ADP/ATP ratio for *S. solfataricus* M16 (polyP+) under 0.5 and 2 mM Cu^2+^ stress, respectively. In light blue is the ADP/ATP ratio for *S. solfataricus* M16-PPX strain (polyP–). Measurements represent the average of three biological replicates, with error bars indicating standard deviations. Data were analyzed using a two-way ANOVA, followed by post hoc multiple comparison tests: Bonferroni’s test was applied for paired comparisons, while Tukey’s test was used for independent group comparisons. Statistical significance is indicated as follows: * = *p* ≤ 0.05, ** = *p* ≤ 0.01 (Bonferroni’s test); # = *p* ≤ 0.05, ## = *p* ≤ 0.01 (Tukey’s test).

Previous studies have reported that *S. solfataricus* contains approximately 30 nmol of Pi per mg of protein [12], but how the polyP is degraded under copper stress has yet to be reported. To address this, *S. solfataricus* cultures were grown to the early stationary phase to ensure maximum polyP accumulation. At this point, cultures were exposed to varying concentrations of CuSO_4_, and samples were collected at different time points following the stress. PolyP was then extracted and quantified (Section 2). The results of polyP degradation under copper stress are shown in Figure 1A.

The data suggest that polyP degradation are dependent on copper concentration, with more pronounced and prolonged degradation observed at higher copper levels (Figure 1A). Specifically, under 0.5 mM Cu^2+^ stress, polyP degradation ceased 30 min post-stress, and the polyP levels remained below their initial values even 24 h later. At 2 mM Cu^2+^ (MIC Value) [12], polyP degradation was significantly greater than at 0.5 mM and persisted for a much longer duration. Similarly to the 0.5 mM condition, the polyP levels did not recover to their initial values even after 24 h of exposure. The differences in polyP degradation between the control and 2 mM Cu^2+^ became statistically significant at 2 h post-stress, as shown in Figure S1, while the 0.5 mM Cu^2+^ condition did not show significant differences.

### 3.2. Energy Metabolism Under Copper Stress in the Presence or Absence of polyP

Given the relationship between polyP and ATP levels, it was crucial to investigate the ADP/ATP ratio in both strains. To this end, cultures were grown to the early stationary phase, and D-arabinose was added to induce the expression of recombinant PPX in the M16-PPX strain, thereby generating cultures with (M16) and without (M16-PPX) polyP. Three hours post-induction, the cultures were subjected to varying concentrations of copper stress. Aliquots were collected at specific time points following the stress, and the ADP/ATP ratio was subsequently measured. The results are presented in Figure 1B.

The results demonstrate distinct responses to copper stress between the two strains. The M16 strain (polyP+) exhibits a decrease in the ADP/ATP ratio at both Cu^2+^ concentrations. In contrast, the M16-PPX strain (polyP-) does not show a significant reduction in the ADP/ATP ratio; instead, a non-significant increase is observed 2 h post-stress, followed by a significant decrease at 4 h, seemingly returning the ADP/ATP ratio to its initial level. Notably, a statistically significant difference between the two strains became apparent as early as 2 h post-stress, likely due to the absence of polyP in the M16-PPX strain.

### 3.3. Protein Aggregation Under Copper Stress in the Presence or Absence of polyP

**Figure 2:**
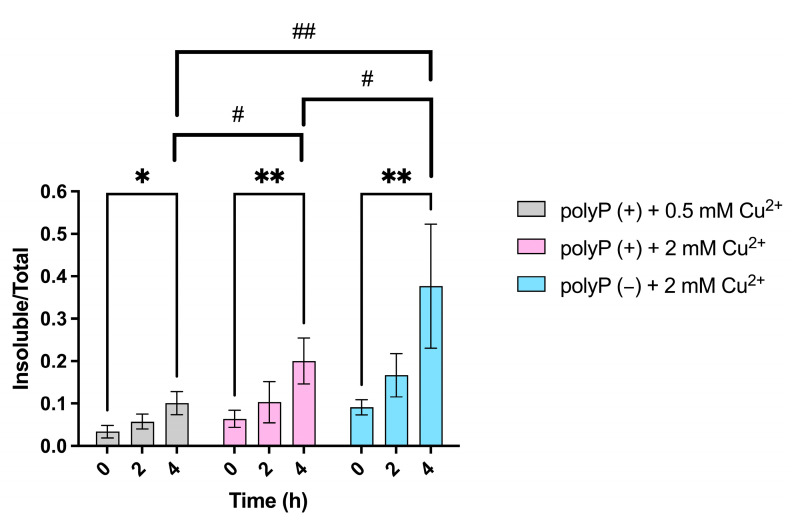
Ratio of insoluble proteins in *S. solfataricus* at different copper concentrations in the presence or absence of polyP. In gray and pink is the ADP/ATP ratio for *S. solfataricus* M16 (polyP+) under 0.5 and 2 mM Cu^2+^ stress, respectively. In light blue is the ADP/ATP ratio for *S. solfataricus* M16-PPX strain (polyP−). Measurements represent the average of three biological replicates, with error bars indicating standard deviations. Data were analyzed using a two-way ANOVA, followed by post hoc multiple comparison tests: Bonferroni’s test was applied for paired comparisons, while Tukey’s test was used for independent group comparisons. Statistical significance is indicated as follows: * = *p* ≤ 0.05, ** = *p* ≤ 0.01 (Bonferroni’s test); # = *p* ≤ 0.05, ## = *p* ≤ 0.01 (Tukey’s test).

*E. coli* cells deficient in polyP exhibit increased protein precipitation due to the inorganic chaperone function of polyP [4,5,16,17,18,20,21,22,23,24,25,26]. To determine whether a similar phenomenon occurs in *S. solfataricus* M16 (polyP+) and M16-PPX (polyP−) strains, soluble and insoluble proteins were extracted from cultures subjected to different copper concentrations. The results can be seen in Figure 2.

The ratio of insoluble proteins (as detailed in the Materials and Methods) shows statistically significant increases in the *S. solfataricus* M16 (polyP+) strain under both 0.5 mM Cu^2+^ and 2 mM Cu^2+^ stress conditions. Notably, a direct comparison of the ratios after 4 h of stress reveals a significantly higher ratio in samples exposed to 2 mM Cu^2+^ (MIC value) (Figure 2).

**Figure 3:**
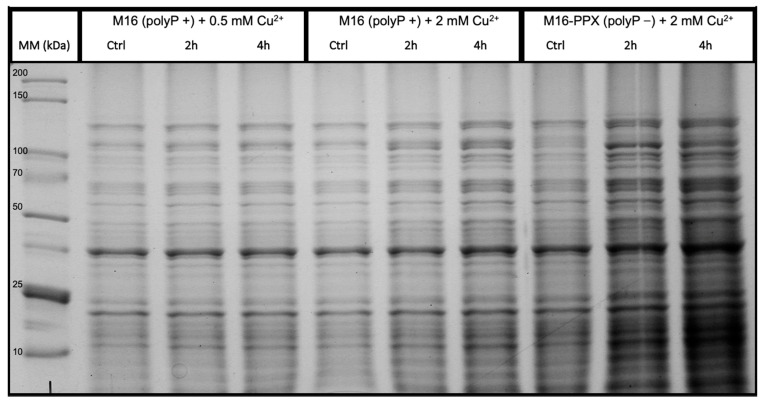
Protein precipitation by the presence of copper is more intense in polyP (−) strain. SDS-PAGE gel with insoluble protein extracted from *S. solfataricus* M16 (polyP+) compared with 0.5 and 2 mM Cu^2+^ stress, and *S. solfataricus* (polyP−) in the presence of 2 mM Cu^2+^. In total, 10 µL of protein suspensions was loaded in each line and stained with Coomassie blue.

The M16-PPX strain (polyP−) demonstrates statistically significant differences in the ratio of insoluble proteins at 4 h post-stress. Moreover, significant differences were observed when comparing the ratio of insoluble proteins between the M16-PPX (polyP−) and M16 (polyP+) strains after 4 h of exposure to 2 mM Cu^2+^. These differences were further confirmed by SDS-PAGE analysis, as shown in Figure 3, where 10 µL of insoluble protein extracts was loaded.

The protein extracts from *S. solfataricus* M16 (polyP+) exposed to 0.5 mM Cu^2+^ showed no apparent increase in protein concentration between the control and 4 h of exposure. However, when this strain was exposed to 2 mM Cu^2+^, there was a noticeable increase in the concentration of insoluble proteins 4 h post-stress, as illustrated in Figure 3. In contrast, the M16-PPX (polyP−) strain exhibited a significantly higher increase in insoluble protein concentration when exposed to 2 mM Cu^2+^ compared to the M16 (polyP+) strain. This corroborates the results seen in Figure 2, where the ratio of insoluble proteins is notably higher in the polyP(−) strain, with statistically significant differences observed between the polyP(+) and polyP(−) strains.

### 3.4. Survival Assay

**Figure 4:**
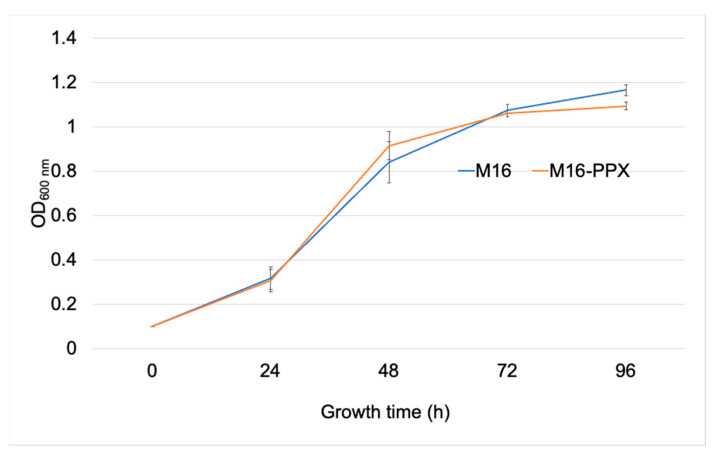
The growth of both strains is not affected by 4 h stress at the MIC of copper. Blue curve for M16 (polyP+) strain; the orange curve for M16-PPX (polyP−) strain. Measurements are the average of three biological replicates. The error bars represent the standard deviations.

Given the possible differential effects of 2 mM Cu^2+^ (MIC value) on the M16 (polyP+) and M16-PPX (polyP−) strains due to the presence or absence of polyP, it was important to assess the growth of these cultures after stress. Both strains were cultivated to the early stationary phase, followed by the addition of D-arabinose to induce *ppx* expression in the M16-PPX strain. After 3 h of induction, complete degradation of polyP occurs in M16-PPX. Subsequently, 2 mM of CuSO_4_ was added to both cultures. Four hours post-exposure, cells were washed three times to remove residual copper and then resuspended in fresh medium at an initial optical density (OD_600 nm_) of 0.02, and growth was measured every 24 h, as seen in Figure 4. The growth curves from both M16 (polyP+) and M16-PPX (polyP−) did not show any difference after 4 h of copper stress. This indicates that the absence of polyP does not affect the survival of cells after 4 h of under 2 mM Cu^2+^ stress.

### 3.5. Expression of Stress-Related Genes Under Copper Stress in the Presence or Absence of polyP

**Figure 5:**
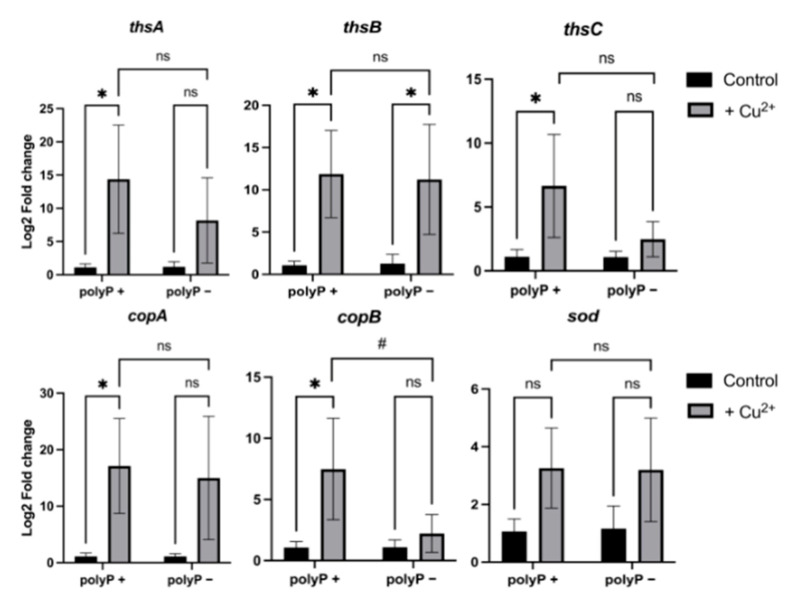
Changes in the transcriptional expression of stress-related genes after 4 h of copper stress in *S. solfataricus* M16 (polyP+) and M16-PPX (polyP−) via qPCR. Measurements represent the average of three biological replicates, with error bars indicating standard deviations. Data were analyzed using a two-way ANOVA, followed by post hoc multiple comparison tests: Bonferroni’s test was applied for paired comparisons, while Tukey’s test was used for independent group comparisons. Statistical significance is indicated as follows: ns = not significant; * = *p* ≤ 0.05 (Bonferroni’s test) and ns = not significant; # = *p* ≤ 0.05 (Tukey’s test).

To assess the expression of stress-related genes under copper stress in the presence or absence of polyP, total RNA was extracted, and the expression levels of chaperonins, chaperones, copper resistance genes, polyphosphate metabolism genes, and superoxide dismutase were quantified by qPCR. Given the findings on protein precipitation, the expression of these genes was specifically evaluated after 4 h of copper stress, as shown in Figure 5.

Figure 5 presents the transcriptional analysis of the three thermosome subunits in *S. solfataricus* (*thsA*, *thsB*, and *thsC*), showing a pronounced overexpression of all subunits in the M16 (polyP+) strain following copper stress. In contrast, the M16-PPX (polyP−) strain displays overexpression of the alpha and beta subunits, with statistical significance observed only for the beta subunit. Figure 5 also illustrates the transcriptional levels of copper resistance determinants in the M16 (polyP+) and M16-PPX (polyP−) strains. In the presence of polyP, *copA* is significantly overexpressed, whereas in the polyP-deficient strain, *copA* expression, although elevated, does not reach statistical significance. A similar pattern was seen for *copB*, which is robustly overexpressed in the M16 (polyP+) strain, with a significantly higher expression level compared to the M16-PPX (polyP−) strain. Strikingly, superoxide dismutase (*sod*) did not exhibit significant differences in expression between the two strains, despite a slight overall increase observed following 4 h of exposure to 2 mM Cu^2+^ (MIC value).

The transcriptional levels of *S. solfataricus* chaperones, including prefoldins (*pfdA* and *pfdB*) and heat shock protein 20 (*hsp20*), were analyzed, and the results are presented in Figure S2. The expression levels of *pfdA*, *pfdB*, and *hsp20* showed no significant differences between the control and 4 h post-copper stress conditions, although a slight increase in expression was observed for both strains. Notably, the M16-PPX (polyP−) strain exhibited significant overexpression of the *ppx* gene, a response absent in the M16 (polyP+) strain. A statistically significant difference in *ppx* expression between the two strains was observed 4 h post-stress, as shown in Figure S2.

## 4. Discussion

PolyP degradation has not been previously characterized in *S. solfataricus*. However, this process was extensively studied in other archaea, such as *Sulfuracidifex metallicus* and *Metallosphaera sedula* [9,11]. PolyP degradation in *S. solfataricus* depends on the copper concentration, being higher and longer when the organism is exposed to bigger copper concentrations. The same phenomenon was reported in *S. metallicus,* in which 100 mM of CuSO_4_ provoked higher polyP degradation than 10 mM [9]. The difference between the degradation in *S. solfataricus* and *S. metallicus* is how fast it happens. While in *S. solfataricus* polyP degradation can last until 2 h, in *S. metallicus*, it lasts for only nearly 15 min [9].

On the contrary, in *M. sedula*, polyP degradation is much slower, like in *S. solfataricus*, lasting 1 h when cells are stressed with 8 mM of copper (MIC value) [11]. Across these extremophilic archaea, including *S. solfataricus*, copper stress does not induce complete polyP degradation. Instead, a residual pool of polyP remains within the cells, allowing the polymer to continue fulfilling its intracellular functions, such as its role as an inorganic molecular chaperone or its role as an energy source [1,4,5,16,17,18,20,21,22,23,24,25,26]. These findings suggest that polyP also plays an important role in copper resistance in *S. solfataricus*. In these thermoacidophilic microorganisms, CuSO_4_ is the copper source commonly used in experimental studies, which is why it was selected for our research. While other copper salts, such as CuCl_2_, could have been tested, they were not considered due to the inhibitory effects of chloride ions on the growth of acidophilic microorganisms frequently utilized in biomining processes [52].

Given the established relationship between polyP and energy metabolism, the ADP/ATP ratio was measured (Figure 1B). Notable differences were observed between the two strains post-stress. In the M16 (polyP+) strain, the ADP/ATP ratio decreased, indicating a higher ATP concentration relative to ADP. In contrast, the M16-PPX (polyP−) strain exhibited a slight, though not statistically significant, increase in the ADP/ATP ratio 2 h post-stress, followed by a decrease at 4 h, returning to initial levels. These differences may be attributed to the presence or absence of polyP in the M16 and M16-PPX strains, respectively. Before the ratio measurement, cultures were induced with D-arabinose (Materials and Methods) to deplete polyP in the M16-PPX strain. In the M16 strain, polyP remained intact at the onset of copper stress. PolyP degradation started upon stress exposure, releasing Pi into the cell, which could be utilized for copper ion chelation or ATP synthesis via ATP synthase. This could explain the decrease in the ADP/ATP ratio. Conversely, in the M16-PPX strain, polyP depletion following D-arabinose induction meant that after copper stress, the cells lacked this resource. As a result, copper expulsion relied solely on CopA and CopB, likely leading to increased ATP consumption and a corresponding shift in the ADP/ATP ratio. This highlights the crucial role polyP plays in energy metabolism during stress, as its degradation not only releases Pi but also helps mitigate the energy demands imposed by metal detoxification processes. Without polyP, energy metabolism becomes less efficient, further emphasizing the importance of polyP in energy regulation under stressful conditions.

Considering that polyP degradation persists for up to 2 h following copper stress, protein precipitation was analyzed in the presence and absence of polyP at 2 and 4 h post-stress. In the M16 (polyP+) strain, the ratio of insoluble proteins (as detailed in the Materials and Methods) exhibited a slight but statistically significant increase under 0.5 mM of Cu^2+^ stress. However, at 2 mM Cu^2+^, a marked increase in protein precipitation was observed after 4 h of exposure. Similarly, in the M16-PPX (polyP−) strain, a significant rise in the ratio of insoluble proteins was detected following 4 h of copper stress. A direct comparison of M16 (polyP+) exposed to 0.5 and 2 mM Cu^2+^ revealed a higher ratio of insoluble proteins at 2 mM Cu^2+^. Interestingly, when comparing the polyP+ and polyP− strains, significant differences in the protein precipitation ratios were observed after 4 h of metal exposure. These findings suggest that in the absence of polyP, its function as an inorganic chaperone is likely compromised, contributing to the elevated levels of protein aggregation observed. This suggests that polyP may play an important role in mitigating protein aggregation under high copper concentrations.

The observed differences in energy metabolism and the high amounts of precipitated proteins in both strains strongly enticed us to conduct a survival assay to evaluate their growth response (Figure 4). Interestingly, no significant differences were detected in the growth curves, even after 4 h of copper exposure, despite the metabolic differences between M16 (polyP+) and M16-PPX (polyP−) strains. This suggests that, although energy metabolism and protein precipitation are differentially affected by the presence or absence of polyP, these changes do not affect survival or growth under the tested conditions.

Given the significant amount of protein precipitation observed in Figure 2 and Figure 3, the expression of various stress response genes was evaluated (Figure 5 and Figure S2). This analysis included chaperonins and chaperones in *S. solfataricus*, including the three thermosome subunits (chaperonins), both prefoldin subunits, and Hsp20 (chaperones). In the M16 (polyP+) strain, all three thermosome subunits were overexpressed (Figure 5), whereas in the M16-PPX (polyP−) strain, only the ThsB subunit exhibited overexpression. No statistically significant differences were observed between the two strains after the 4 h copper stress, although the expression of *thsC* appeared higher in the polyP+ strain.

Among the chaperones analyzed (Figure S2), no significant overexpression was detected, although slight increases were observed in both strains following stress exposure. Previous studies in *E. coli* have shown that, under oxidative stress and in the absence of polyP, chaperones were overexpressed as early as 10 min after stress, a phenomenon attributed to polyP’s role as an inorganic chaperone [16]. The lack of chaperone and chaperonin overexpression in the polyP− strain compared to the polyP+ strain may be due to the timing of the expression measurements. This observation suggests that polyP’s function as an inorganic chaperone may be critical during the early stages of the stress response, even as it is being degraded by PPX (Figure 1A). This protective role during the initial phases of stress becomes more apparent later, as evidenced by reduced protein aggregation in the M16 (polyP+) strain compared to the M16-PPX (polyP−) strain (Figure 2 and Figure 3).

Strikingly, despite the 2 mM Cu^2+^ stress (MIC value) and the high amount of insoluble proteins seen in both strains after 4 h of stress (Figure 2 and Figure 3), no significant expression of *sod* was detected (Figure 5). This suggests that the stress caused by copper may not be primarily oxidative. Previous studies in *E. coli* have reported that copper can also induce protein precipitation by interacting with amino acids on the protein surface, specifically cysteines and histidines, which can disrupt the protein’s three-dimensional conformation, leading to precipitation [51].

Copper resistance determinants such as CopA and CopB were also measured by qPCR (Figure 5). While in the M16 (polyP+) strain there is the overexpression of *copA* and *copB*, in the M16-PPX (polyP−) strain there was no overexpression, although there were elevated levels of *copA.* These genes codify transporters crucial for copper resistance, which use ATP for copper expulsion [33]. As previously mentioned, in the M16-PPX strain, the absence of polyP and the observed changes in energy metabolism suggest that copper resistance would rely primarily on CopA and CopB. However, no significant overexpression was observed for *copA*, despite a slight increase, and *copB* showed no increase at all. This is particularly notable given that CopB has been associated with high copper concentrations in the growth medium [33].

Finally, the expression of *ppx* was also measured by qPCR. As shown in Figure S2, no overexpression was seen in the M16 (polyP+) strain, even under 2 mM Cu^2+^ stress, despite the reported role of PPX in polyP degradation under stress conditions [1,2,3,4,5,6,7]. On the contrary, the overexpression of *ppx* in the M16-PPX (polyP−) strain was expected, as this strain, as described in the Materials and Methods, was induced with D-arabinose to express the PPX enzyme.

## 5. Conclusions

Our study highlights the critical role of polyphosphate in the response of *S. solfataricus* to copper stress, with polyP degradation directly linked to copper concentration. On the other hand, a decrease in the ADP/ATP ratio was seen in the polyP (+) strain, suggesting that polyP contributes to maintaining cellular energy homeostasis under stress by providing inorganic phosphate for ATP synthesis. In contrast, the polyP (−) strain exhibited altered energy dynamics, likely due to the lack of polyP.

Protein aggregation was notably higher in the absence of polyP, suggesting that polyP may act as an inorganic chaperone in archaea, protecting proteins from stress-induced denaturation. Interestingly, no significant overexpression of chaperonins or chaperones was observed in the polyP (−) strain 4 h after copper stress compared to the expression levels observed in the polyP (+) strain. This implies that polyP’s role as an inorganic chaperone may be most critical during the early stages of stress. The protective function of polyP may take time to manifest, as indicated by the results of our protein aggregation experiments. These findings provide new insights into polyP’s potential chaperone role. Furthermore, the lack of sod overexpression in both strains suggests that the copper stress response in *S. solfataricus* may not primarily involve oxidative stress. Instead, it could be driven by direct interactions between copper and proteins.

In summary, this study provides novel insights into the role of polyP in the copper stress response of *S. solfataricus*, functioning both as an energy reserve and as a protection against protein aggregation by acting as an inorganic chaperone. To our knowledge, these findings are the first to address the role of polyP as an inorganic chaperone in archaea. Further studies will be required to explore the interesting role of polyP in other extremophilic archaea.

## Data Availability

The original contributions presented in this study are included in the article/Supplementary Materials. Further inquiries can be directed to the corresponding author.

## Supplementary Materials

The following supporting information can be downloaded at: https://www.mdpi.com/article/10.3390/microorganisms12122627/s1, Figure S1: Statistical analysis of polyP degradation under stress with different copper concentrations; Figure S2: Changes in the transcriptional expression of stress-related genes after a 4 h copper stress in *S. solfataricus* M16 (polyP+) and M16-PPX (polyP−) via qPCR; Table S1: Primers used in this study.
