# Supplementary material for: Role of Polyphosphate as an Inorganic Chaperone to Prevent Protein Aggregation Under Copper Stress in Saccharolobus solfataricus
Source: Microorganisms. 2024 Dec 18;12(12):2627. doi: 10.3390/microorganisms12122627 (PMC11677633; doi:10.3390/microorganisms12122627)
Supplement: Supplementary file 1 [file microorganisms-12-02627-s001.zip › microorganisms-3340023-supplementary.pdf]

Supplementary Material

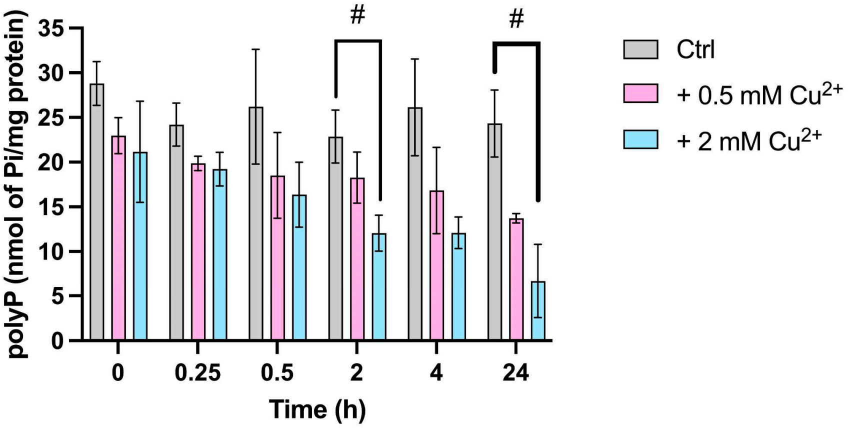

Figure S1. Statistical analysis of polyP degradation under stress with different copper concentrations. Two-way ANOVA was performed, followed by Tukey's post hoc test for multiple comparisons. Three biological replicates were used, and data are presented as the mean of these replicates. Error bars represent standard deviations. # = $p \leq 0.05$.

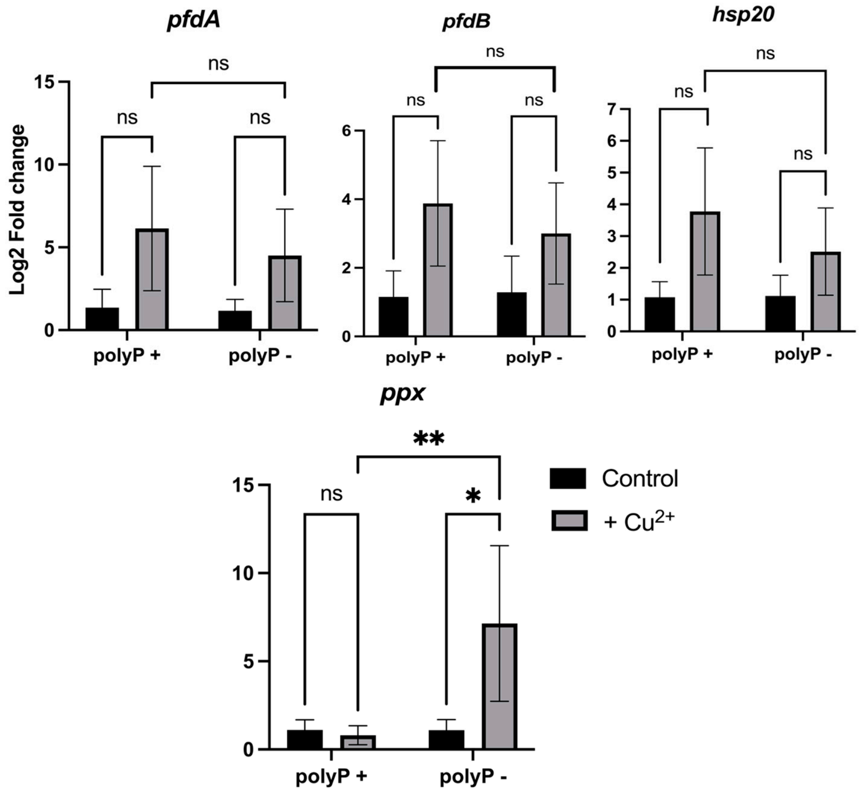

Figure S2. Changes in the transcriptional expression of stress-related genes after a 4 h copper stress in *S. solfataricus* M16 (polyP +) and M16-PPX (polyP -) via qPCR. Measurements represent the average of three biological replicates, with error bars indicating standard deviations. Data were analyzed using a two-way ANOVA, followed by post hoc multiple comparison tests: Bonferroni's test was applied for paired comparisons, while Tukey's test was used for independent group comparisons. Statistical significance is indicated as follows: ns = not significant; * = $p \leq 0.05$ (Bonferroni's test) and ns = not significant; # = $p \leq 0.05$ (Tukey's test).

Table S1. Primers used in this study

Primer	Sequence
23S Fw	ggtcttgcctttcgtcctga
23S Rv	tcggtttccctatgccttcg
thsA Fw	aagtgtgggaggtaaggagc
thsA Rv	tcccgcctatgacatctacac
thsB Fw	tggaaggtgcgtaaaatggc
thsB Rv	ttcagcgcctcttcaattgc
thsC Fw	ggtggtggagcagtagaaga
thsC Rv	ccagttgcgtgtttgtgtct
pfdA Fw	atgcagaagtggacctagactc
pfdA Rv	ttcaccttgttgtctcctggc
pfdB Fw	acctccagaagttcaagctca
pfdB Rv	gcattgagaacggataactcttc
hsp20 Fw	cgcgaaccattagctgatgt
hsp20 Rv	cttagccgccttctcgtcta
copA Fw	tcacaacacattttacgccga
copA Rv	ctctctcccgccctaacaat
copB Fw	aggtattttctcccctggct
copB Rv	cattgcacttatcggctgga
sod Fw	atggacacaagttacacgcc
sod Rv	ccagtcccgggtagtgaatt
ppx Fw	tcaatgggacgacatgatt
ppx Rv	gcagaggaagcgacatagaa
